# Limbic Justice—Amygdala Involvement in Immediate Rejection in the Ultimatum Game

**DOI:** 10.1371/journal.pbio.1001054

**Published:** 2011-05-03

**Authors:** Katarina Gospic, Erik Mohlin, Peter Fransson, Predrag Petrovic, Magnus Johannesson, Martin Ingvar

**Affiliations:** 1 MR Research Center and Osher Center for Integrative Medicine, Department of Clinical Neuroscience, Karolinska Institute, Stockholm, Sweden; 2 Department of Economics, University College London, London, United Kingdom; 3 Department of Economics, Stockholm School of Economics, Stockholm, Sweden; University of Zurich, Switzerland

## Abstract

Imaging studies have revealed a putative neural account of emotional bias in decision making. However, it has been difficult in previous studies to identify the causal role of the different sub-regions involved in decision making. The Ultimatum Game (UG) is a game to study the punishment of norm-violating behavior. In a previous influential paper on UG it was suggested that frontal insular cortex has a pivotal role in the rejection response. This view has not been reconciled with a vast literature that attributes a crucial role in emotional decision making to a subcortical structure (i.e., amygdala). In this study we propose an anatomy-informed model that may join these views. We also present a design that detects the functional anatomical response to unfair proposals in a subcortical network that mediates rapid reactive responses. We used a functional MRI paradigm to study the early components of decision making and challenged our paradigm with the introduction of a pharmacological intervention to perturb the elicited behavioral and neural response. Benzodiazepine treatment decreased the rejection rate (from 37.6% to 19.0%) concomitantly with a diminished amygdala response to unfair proposals, and this in spite of an unchanged feeling of unfairness and unchanged insular response. In the control group, rejection was directly linked to an increase in amygdala activity. These results allow a functional anatomical detection of the early neural components of rejection associated with the initial reactive emotional response. Thus, the act of immediate rejection seems to be mediated by the limbic system and is not solely driven by cortical processes, as previously suggested. Our results also prompt an ethical discussion as we demonstrated that a commonly used drug influences core functions in the human brain that underlie individual autonomy and economic decision making.

## Author Summary

It is well-established that emotions influence decision making. One way of studying this relationship is the Ultimatum Game, which has revealed that subjects punish unfair behavior in others in spite of receiving a concomitant economic loss. Previous brain imaging studies have suggested that this decision to punish involves complex cortical processing. However, punishment also involves an instant aggressive emotional response, a behavior often linked to subcortical structures such as the amygdala. In this study, we present a model that joins these views. We designed a paradigm that allows us to measure the activity of subcortical brain regions during decision making in the Ultimatum Game, while at the same time using a pharmacological approach that can suppress emotional responses and amygdala activity. The pharmacological treatment made subjects punish unfair behavior less, and decreased brain activity in the amygdala in response to unfair proposals, without changing the subjects' feeling of unfairness. In the control group, punishment was directly linked to an increase in amygdala activity. Thus, immediate punishment of unfair behavior involves the amygdala and is not solely driven by cortical processes, as previously suggested. Our results show that a commonly used drug influences autonomy and decision making, which may have ethical implications for its use.

## Introduction

Research within behavioral economics and psychology has demonstrated that human decisions are based on more dimensions than simply maximization of monetary reward [1]–[3]. One important factor with prominent impact on decision making is the influence of emotional processes [4]. Emotional processes include both emotional responses [5] and feeling states [6]. Emotional responses are rapid and automatic in order to meet the demands for fast contextual adaptation. On the other hand, the representation of feeling states and the regulatory control of emotions reflect a slower adjustment to long-term considerations and goals [6]. Recently, brain imaging studies have shown that emotional systems are active in decision making [2],[7]–[9]. However, it is difficult to identify the causal role of the different sub-regions involved in decision making based on these correlational studies.

A human universal in social cooperation is the tendency to respond with immediate aggression upon perceived threat or unfairness [10]. Evolution seems to have favored the act of punishing those who violate perceived norms of the group [11]. A suitable paradigm to study the punishment of norm-violating behavior is the Ultimatum Game (UG) [12]. In the UG, a proposer suggests a way to divide a fixed sum of money. The responder has to accept or reject the proposal. If the responder accepts the proposal, the suggested split is realized. If the responder rejects the offer, neither of the two gets anything. Proposers often offer an even split, and unfair offers are frequently rejected; offers of ≤20% are rejected roughly half of the time [3]. These findings are robust with respect to learning effects, stake size, and other manipulations [3]. Although both individual genetic traits and cultural variation influence the response pattern, the general propensity to punish norm violators seems to be universal [13],[14].

While there have been previous attempts within the field to include anatomical information in the behaviorally validated theoretical models of decision making, a mechanistic explanation of how emotions actually influence choice is still missing [15]. Existing interdisciplinary economic models are incomplete [16] in that the biological framework is not fully defined. Several studies have suggested that emotional theory may add important information for choice behavior [1],[2]. The evolution of the frontal lobes in humans has extended the ability for long-term reasoning [17]. In a pioneering paper Sanfey et al. [7] suggested that the forebrain network (anterior insula, dorsolateral prefrontal cortex [dlPFC], and anterior cingulate cortex [ACC]) has a pivotal role in the rejection response in the UG [7]. This result is at variance with a vast literature that attributes a crucial role in emotional decision making to a subcortical structure (i.e., the amygdala) [2],[4],[18]. Here we propose an anatomy-informed model that may link these seemingly opposing views. Gläscher et al. [19] proposed that intuitive decisions could be considered as cognitively model-free reinforcement learning (RL), whereas deliberate choices could be viewed as model-based RL. By combining computational learning models with functional imaging data they found that model-free RL was associated with subcortical processing and model-based RL was linked to cortical processing. They suggested that decision making involves at least two neural networks that seem to have distinct neural correlates. Thus, both cortical and subcortical levels may influence the final behavior in either direction in the UG, and the major difference is that the cortical level has a richer representation of future outcomes of a decision [17],[19].

In the UG, the payoff-maximizing strategy for the responder (individual level) is to accept all offers, and reciprocally for the proposer it is to make the smallest possible offer [12]. The UG demands a simple and rapid yes/no answer, but the underlying reason for a response is complex and extends beyond payoff maximization and includes influences from social interactions. For example, the responder needs to consider: social hierarchy, tit-for-tat, preparing for the next encounter, maintenance of social norms, reputation building, and avoidance of social rejection.

Short-term unaware responses are instantiated in the subcortical emotion system (e.g., the amygdala) [4],[20],[21], whereas the long-term considerations pertain to frontal cortex and insula [22],[23]. In order to reconcile the paradox in the literature on the UG [7],[24] and decision making [2],[4], we hypothesized that the response in the UG rests on a balance between phylogenetically older structures involved in the automatic reactive emotional response and neocortical areas associated with the neural processing of feeling states, future representation, and regulation of emotions [5],[6],[17].

**Figure 1:**
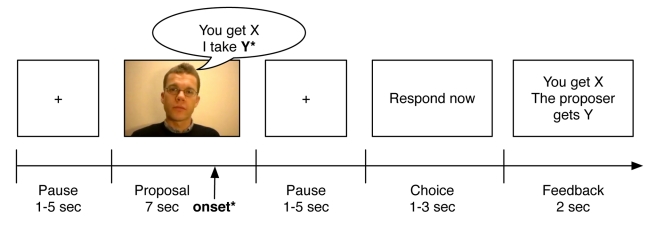
Experimental set-up. Thirty-five subjects were randomly assigned to either the control (placebo pill) or the treatment group (oxazepam, 20 mg orally). One hour after treatment subjects played the UG in the MRI scanner by watching 45 movie clips, each with a different human proposer. The proposals were fair, unfair, or neutral. All proposals had the exact same wording, and the proposer ended the sentence by stating the share that he/she would get. The fMRI onset time was defined as when the last word was spoken, i.e., when the fairness of the proposal could be judged. Subjects were instructed to respond either “yes” or “no” to the fair/unfair proposals and “no” to the neutral proposals. After scanning, subjects rated the fairness of the offers (scale 1–7) [7] and likeability of the proposers' faces (scale 0–100).

Previous imaging studies [7],[24] on decision making in the UG did not specifically aim to separate instant automatic responses from slower affective processes associated with awareness [6]. The immediate responses are likely to be transient and mitigated when emotional regulation sets in [25]. Thus, a prerequisite to detect these responses is to have a strictly defined onset time for when unfairness may be detected. Fast automatic responses in the UG have not previously been captured since the proposals were presented for 6 s [7],[24], thereby not providing a clear definition of the onset time for possible detection of unfairness. Thus, in the previous studies, there is a possibility that the experimental design precluded a detection of early and transient responses. In our experiment, the proposals were given orally in movie clips, and we formulated the UG proposal so as to maintain ambiguity of fairness until the final word of the proposal (i.e., the amount that would be taken by the proposer was spoken last), yielding a well-defined onset time (Figure 1).

As the amygdala is crucial for both the mediation of aggressive responses [26] and of biasing decision making [2],[4], we suggest a parallel between reactive aggression and the behavior associated with rejection. Thus, we hypothesized that the amygdala drives immediate rejection in the UG.

GABA receptors are abundant in the amygdala, and benzodiazepines can potentiate GABA activity, reduce behavioral signs of aggression [26], and decrease amygdala activity in emotional tasks [27],[28]. In the present study, we posited that the benzodiazepine oxazepam (20 mg orally) could inhibit amygdala activity and, thus, change behavior in the UG. We assumed that unfair proposals would generate an amygdala response and increase rejection rate in the non-medicated group, while oxazepam would inhibit this process and therefore reduce the rejection rate in response to unfair proposals, in parallel with a mitigated amygdala response to unfair proposals. Thus, to test whether the amygdala is involved in the rejection of unfair offers, we randomly allocated subjects to a treatment or a placebo group prior to scanning them while playing the UG.

As will be seen, our design allowed for the detection of the functional anatomical response to unfair proposals in a subcortical network that mediates rapid reactive responses. This response was possible to manipulate selectively with a benzodiazepine both on the behavioral and the neural response level.

## Results

### Treatment Decreases Rejection Rate for Unfair Proposals

**Figure 2:**
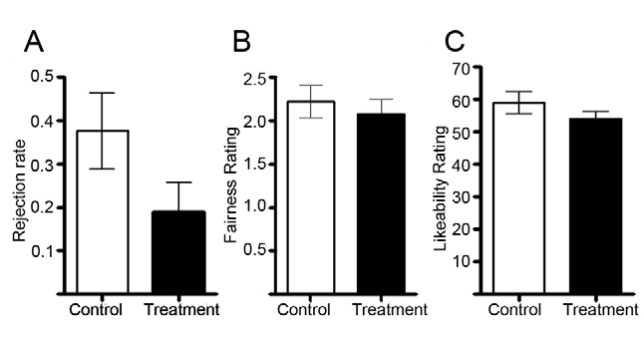
Rejection rate and subjective ratings of fairness and likeability. (A) Treatment with oxazepam (*n = *18) reduced the rejection rate of unfair offers by 49% compared to the control treatment (*n = *17) (Mann-Whitney *U* test, one-tailed, *Z* = 1.722, *p* = 0.049). Rating of fairness for unfair offers (B) and likeability rating of the proposers for rejected/accepted proposals (C) did not change with treatment (fairness, *Z* = 0.658, *p* = 0.51; likeability, *Z* = 0.603, *p* = 0.55). All the ratings were analyzed with the Mann-Whitney *U* test, two-tailed.

In line with previous studies, fair proposals were never rejected in either group [7],[29]. The rejection rate (not controlled for gender) for unfair proposals was significantly lower in the oxazepam group (19.0%; *n = *18) than in the placebo group (37.6%; *n = *17; *p* = 0.0475; Figure 2A). The same comparison but controlling for gender in a probit regression showed borderline significant results (*p* = 0.0675); the predicted rejection rate was 19.5% in the oxazepam group and 36.1% in the placebo group. However, the gender coefficient was insignificant (*p* = 0.278). The treatment-induced drop in the rejection rate was 20 percentage points for men and 15 percentage points for women. Neither the rejection rate nor the effect of treatment on the rejection rate varied significantly between men and women (see Text S1 for details).

### Main Effect of Fairness Confirms Previous Literature

**Figure 3:**
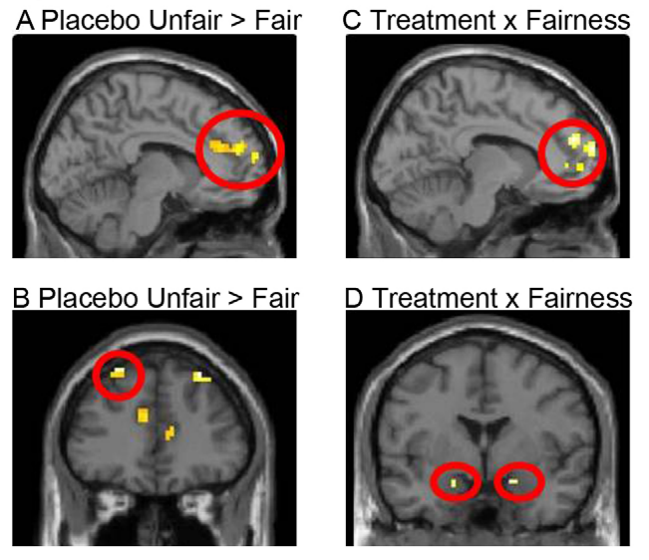
fMRI data related to unfair proposals. In the placebo group (*n = *18), we replicated data from Sanfey et al. [7] in that unfair proposals elicited activity in (A) right ACC (Montreal Neurological Institute space coordinates (x, y, z): [9 48 24], *Z = *3.15, *p<*0.001, uncorrected) and (B) bilateral dlPFC (Montreal Neurological Institute space coordinates (*x*, *y*, *z*): left, [−24 36 54], *Z = *4.04, *p<*0.001, uncorrected; right, [30 36 51], *Z = *4.04, *p<*0.001, uncorrected). There were more expressed responses in the placebo group (interaction placebo _unfair−fair proposals_>oxazepam _unfair−fair proposals_) with an increased activation in (C) left mPFC ([−6 66 18], *Z = *3.77, *p<*0.05, cluster level corrected) and ACC ([9 48 24], *Z = *3.57, *p<*0.05, cluster level corrected) and (D) bilateral amygdala (left, [−18 −6 −18], *Z = *3.25, *p<*0.05, corrected; right, [18 0 −18], *Z = *3.03, *p<*0.05, corrected). Treatment with oxazepam (*n = *18) lowered the neural responses related to unfair proposals.

The functional MRI (fMRI) contrast of unfair versus fair in the placebo group essentially confirmed the results from Sanfey et al. [7] (Figure 3A and 3B). The corresponding contrast in the oxazepam group showed a subsignificant activation in the right insula (see Figure S1 and Table S1).

### Treatment Suppresses Neural Activity Related to Rejection

**Figure 4:**
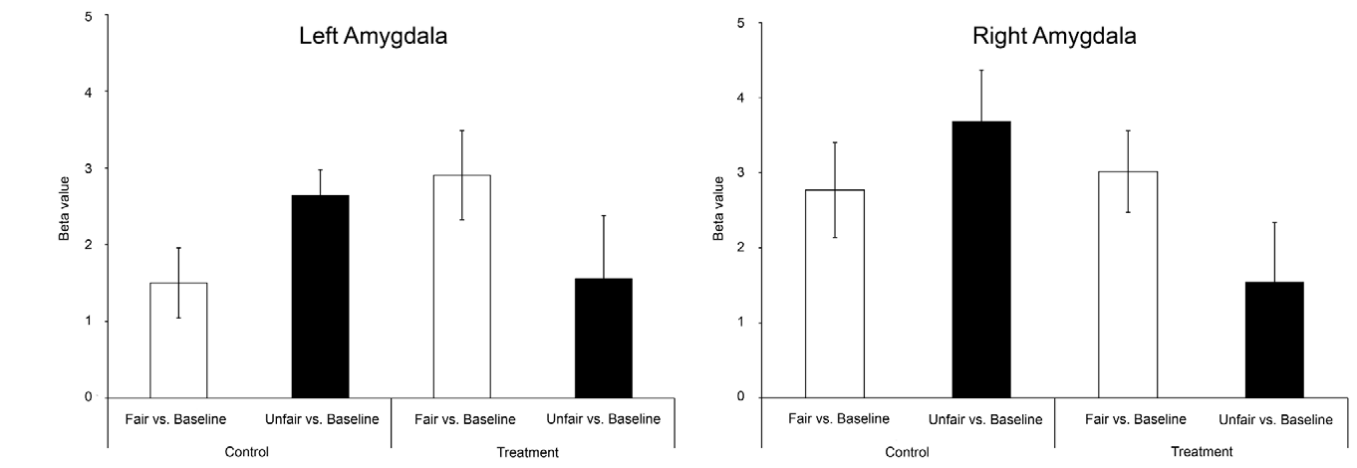
Parameter estimates in the right and left amygdala. Left amygdala: placebo fair, 1.50±0.46 (mean ± standard error of the mean); placebo unfair, 2.64±0.34; oxazepam fair, 2.91±0.58; oxazepam unfair, 1.56±0.82. Right amygdala: placebo fair, 2.77±0.63; placebo unfair, 3.69±0.68; oxazepam fair, 3.02±0.54; oxazepam unfair, 1.55±0.79.

Given that oxazepam inhibits rejection of unfair offers, the interesting contrast is the interaction that probes for changes in response to unfairness with or without oxazepam (placebo _unfair−fair proposals_>oxazepam _unfair−fair proposals_). We confirmed our primary hypothesis that the amygdala was relatively more activated in the placebo group than in the oxazepam group for unfair offers (left amygdala: Montreal Neurological Institute space coordinates (x, y, z) [−18 −6 −18], *Z* = 3.25, *p* = 0.05, corrected; right amygdala: [18 0 −18], *Z* = 3.03, *p<*0.05, corrected; Figure 3D). The amygdala response for the different conditions is also visualized in Figure 4, where we show that both groups have similar amygdala activation patterns in response to the fair condition; however, unfairness up-regulates the amygdala response in the placebo condition while oxazepam reduces the amygdala response in the treatment group.

Moreover, the extended fMRI analysis revealed interaction differences in medial prefrontal cortex (mPFC) ([−6 66 18], *Z* = 3.77, *p<*0.05, cluster level corrected) and right ACC ([9 48 24], *Z* = 3.57, *p<*0.05, cluster level corrected) (Figure 3C). Thus, subjects who were treated with oxazepam had a diminished activation in a subset of regions in the neural network normally activated by unfair proposals [7]. The change in rejection rate induced by oxazepam did not result in any significant effects in the dlPFC or the insula, two areas that have previously been linked to the rejection of unfair offers in the UG [7],[29]. Given the previous data in the literature, we extended the analyses and performed post hoc specific searches with a single-region-of-interest approach in the dlPFC and the insula. We noted effects in the interaction also in these regions (see Text S1). Importantly, the general cerebral response in the decision making task was unaltered by the drug (see Text S1 and Figure S2).

### Rejection of Unfair Proposals Increases Amygdala Activity in the Placebo Group

**Figure 5:**
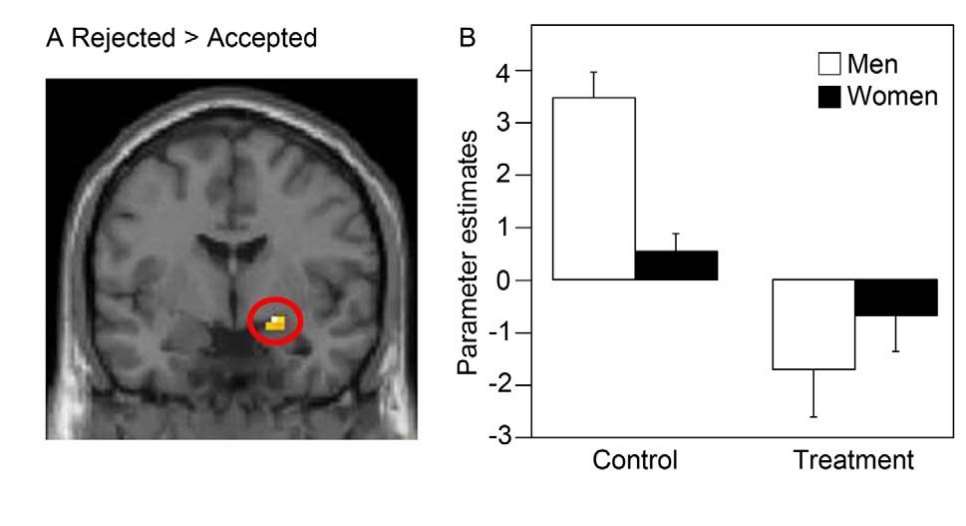
fMRI results related to rejection of an unfair proposal and sex difference. (A) The rejection of an unfair proposal was associated with a higher activity in the right amygdala ([21 −3 −12], *Z* = 3.50, *p<*0.05, corrected) in the placebo group (*n = *6). (B) The difference between sexes for unfair versus fair proposals in the placebo condition was significant (*t*[15] = 4.30, two-tailed, *p* = 0.001; males, *n = *5; females, *n = *12). There was also a main effect of treatment (*F*[1] = 18.53, *p* = 0.000) and an interaction between gender and treatment for unfair versus fair proposals (*F*[1] = 8.50, *p* = 0.007). Mean ± standard error of the mean for parameter estimates in the right amygdala.

We predicted that increased amygdala activity would correspond to an increased rejection rate also within the placebo group, as the amygdala is known to have a crucial role in decision making [2],[4] and aggression [26]. To test this hypothesis we did a within-subject analysis in placebo subjects that both accepted and rejected unfair proposals (*n = *6) in both experimental sessions. The contrast unfair proposals _rejected_>unfair proposals _accepted_ showed increased amygdala activity ([21 −3 −12], *Z* = 3.50, *p<*0.05, corrected; Figure 5A).

### Males Show a Greater Amygdala Response to Unfair Proposals

As testosterone can increase aggressive behavior [26], we tested whether males drive the reactive amygdala response and hence would show an increased amygdala activity in response to unfairness. To test this hypothesis, we compared amygdala activity between sexes for the contrast placebo _unfair−fair proposals_>oxazepam _unfair−fair proposals_. Strikingly, males (*n = *5) showed a greater right amygdala activity than females (*n = *12) in the placebo condition, while there was no difference between sexes in the oxazepam condition (males, *n = *8; females, *n = *10; Figure 5B). Thus, the interaction sex × treatment was significant (*F*[1] = 8.50, *p* = 0.007). However, it is important to emphasize that the effect seen in the interaction contrast cannot be explained as a gender effect as the result remains significant (left amygdala: *p* = 0.035; right amygdala: *p* = 0.042) even after adjusting for gender (see Text S1).

### No Effects of Treatment on Ratings of Unfairness and Likeability

The subjects treated with oxazepam displayed a decreased rejection rate to unfair proposals (Figure 2A). In order to probe the possibility that the change in behavior was due to drug-altered perception of unfairness or likeability of the proposer, we compared subjective ratings between groups. Subjects in the oxazepam group had similar perception of unfairness (Mann-Whitney *U* test, two-tailed, *p = *0.5103; Figure 2B) and perceived likeability of the proposers (Mann-Whitney *U* test, two-tailed, *Z = *0.63, *p = *0.5467) as in the control group (Figure 2C). This is in concordance with the finding that there was no difference in insula activity between the groups in unfair versus fair offers (see Figure S1) (the insula is involved in the coding of feeling states [6]). Thus, we found that the observed change in choice behavior between the treatment groups was not explained by an altered feeling of unfairness or insula activity.

## Discussion

We are the first, to our knowledge, to show the functional anatomical response to unfair proposals in a subcortical network for rapid reactive responses. Our results suggest that the act of immediate rejection of unfair proposals is driven by a phylogenetically old structure (the amygdala) and may be viewed as a reactive aggressive response. This finding adds additional information, as previous UG studies have shown involvement of only a cortical network [7],[24]. We propose that the subcortical and the cortical networks can operate separately and that the major differences between the two are that cortical networks have a richer future representation and operate more slowly [17],[19]. Moreover, we demonstrated that the amygdala-driven rejection response was inhibited with oxazepam treatment without affecting the perception of unfairness. This suggests that the GABA system can influence the decision making network via an alteration of the balance between phylogenetically young (prefrontal cortex) and old structures (amygdala).

As timing is crucial for detection of transient responses [30], our design had the necessary elaboration to allow detection of fast automatic emotional responses to unfairness and not only slow components. We observed a clear amygdala activation that is in line with previous studies (with proper onset timing) on emotional bias in decision making [2],[28]. Our study generates two arguments for a causal role of the amygdala in the generation of an *instant* rejection response. First, in the treatment group, the amygdala response was mitigated in conjunction with a decreased rejection rate (as compared with the placebo group). Second, in the within-subjects comparison in the unmedicated group, rejections were associated with increased amygdala activity. In light of this, we question the suggested exclusive causality of the insula in the generation of a rejection response that was derived from the correlation between insula activity and acceptance rate of UG offers [7]. Instead, we propose that the amygdala is involved in instant rejection of unfair UG offers, whereas the insula might be more involved in a late rejection response. Importantly, neither result excludes that separate neural operations can give rise to the same behavior. Moreover, it is important for future research to segregate which responses are related to perception of unfairness and which drive rejection behavior, and how these neural processes interact.

Our data are compatible with the two-level model for decision making [19]. We have pharmacologically manipulated both levels in our study. The effects we observed may be based either on direct pharmacological action or on interaction effects between regions in the decision making circuit. In another manipulation of this circuit Knoch et al. [29] showed that transcranial magnetic stimulation of the dlPFC leads to an increased acceptance rate for unfair proposals in the UG, without changing the perception of unfairness. The authors concluded that dlPFC exclusively drives rejection in response to unfair proposals. We suggest that the interpretation could be modified, as dlPFC does not seem to play a role in low-level model-free decision making [19],[22]. In support of this modified interpretation we note that, in a decision making task where the subject had to remember explicit values supporting the decision, the dlPFC was shown to be crucial [31]. As the prefrontal regions mature over the first years of life, the concept of fairness and theory of mind develop across the same age. The above interpretation predicts that children will not act as adults. Indeed, Takagishi et al. [32] demonstrated that preschoolers do reject unfair offers in spite of having no explicit account of unfairness or theory of mind. Thus, these findings suggest that dlPFC might be sufficient but not necessary for rejection, and support a two-level model for decision making.

Our data provide some additional insights into the role of the prefrontal regions in decision making. We observed a relative increase in rostral ACC/ventromedial PFC for unfair versus fair proposals in the unmedicated versus oxazepam treatment group, but no changes in the dlPFC. We suggest that this treatment-related change in rostral ACC/ventromedial PFC is secondary to reduced amygdala input mirroring a reduction of conflict [33]. However, it is not possible to exclude that the rostral ACC/vetromedial PFC are part of the attentional control system and therefore could be directly modulated by the treatment.

We have shown that the basis for decision making in the UG has underpinnings in several brain regions of different phylogenetic origin, and this underlines the complexity of responses in the UG. Our data suggest that the automaticity driven rejection response has a phylogenetically older representation than the calculated acceptance based on a consciously determined self-optimizing strategy. The amygdala-driven reactive aggressive response generates a behavior that, for example, yields an acceptable splitting of a prey within the group, and such an inequity aversion is seen in children [32]. Thus, automatic individual reactions to detected unfairness seem, to a certain extent, to support the long-term group norms that allow sharing. More developed sharing schemes like formal trade and abstract rule obedience require that each individual can maintain concepts of future effects of present decisions [29],[31]. Such social interactions rest on the development of the human frontal lobe function. We have demonstrated that an anxiolytic drug alters the balance between rapid emotional reactions and reflected-feeling-based decisions. The finding prompts an ethical discussion, as we showed that a commonly used drug influences core functions in the human brain that underlie individual autonomy and economic decision making.

## Materials and Methods

### Subjects

Thirty-five right-handed volunteers with the mean age of 23.7±4.2 y (13 men, 22 women) were included in the study. Subjects were randomly assigned to either group independent of gender (five males and 12 females in the placebo group and eight males and ten females in the oxazepam group) and had no prior or present history of psychiatric illness or neurological disease. All subjects were healthy and took no medications, with the exception of birth control pills and mild allergy medications. All participants gave their informed consent. The study was approved by the local governmental ethics committee in Stockholm, Sweden.

### Stimuli/Ultimatum Game

Each subject was exposed to 45 different movie clips. In each movie there was a different human proposer who made a fair, unfair, or neutral suggestion (see below) on how to split a sum of money. The fair proposals implied an equal split of the money; the proposer said, for example, “You get 50 Swedish crowns, and I take 50 Swedish crowns” (7 Swedish crowns [SEK]≈US$1). The unfair proposals implied that the responder should receive 20% and the proposer 80% of the money, for example, “You get 20 Swedish crowns, and I take 80 Swedish crowns.” The total stakes (e.g., 100 SEK) were deliberately never mentioned, to maintain ambiguity of fairness until the final proposition of the amount that would be awarded the responder was revealed. All proposals had the exact same wording, except for the monetary amounts, since the total stakes varied. Subjects were instructed to respond with either “yes” or “no” to the proposals by pressing a button. In the neutral control condition the subjects were shown films with proposers saying “this is not a proposal,” and subjects were instructed to respond “no” to these.

The three different stake levels yielded a total of seven different kinds of messages. Each subject encountered six 50/50 offers, seven 20/80 offers, five 125/125 offers, five 50/200 offers, four 250/250 offers, three 100/400 offers, and 15 neutral messages. The genders of the proposers were thoroughly balanced (22 males, 23 females).

Before each movie clip the subject was presented with a resting frame containing a hair cross, for a duration that was randomized between 1 and 5 s. Thereafter, a film clip with an offer was presented. The onset times when the proposer finished the sentence were included as regressors of interest (individual regressors for fair, unfair, and no proposals) in the subsequent general linear model analysis of the fMRI analysis. Each movie lasted for 7 s. The clip was followed by a pause, which was again randomized between 1 and 5 s. Thereafter, a frame was shown saying “respond now,” instructing the subject to make a choice. This frame lasted until a choice had been made or maximally 3 s. The onset times when the subject pressed the button for “yes” or “no” were included as a covariate of no interest in the subsequent general linear model analysis of the fMRI analysis. Finally, a frame confirmed the decision, with a sign stating “you got *X* Swedish crowns, your counterpart got *Y* Swedish crowns” (2 s).

### Proposal Films

A total of 92 individuals were filmed while they read each of the messages as explained above. All individuals were filmed under the same conditions, with light from the front, against a white background, and with the eyes located in the middle of the screen while speaking into the camera. The films with 45 of these individuals were kept, and the others were discarded because of low sound quality or because the person did not look into the camera as desired.

### Monetary Reward

Subjects acting as responders were paid 300 SEK for showing up. In addition, three of the 45 movies presented to them were selected at random, and paid out with real money, both for themselves and for the proposer. If the subject had answered “yes” to such a selected proposal, both participants were subsequently paid the corresponding amounts of money. In contrast, if the subject had declined the proposal then neither of the two received any money from that film. This information was given to the subjects before the experiment. The persons acting as proposers on the film clips were given 100 SEK for making the films. They were also subsequently paid their part of the proposals that were drawn randomly, as described for the proposers. Average payment to proposers was 380 SEK, and average payment to responders was 625 SEK.

### Experimental Procedures

Upon arrival, subjects were randomly assigned to either the placebo group (five men, 12 women) or the oxazepam group (eight men, ten women). The subjects in the oxazepam group received 20 mg of the drug. Both treatment and placebo were administered orally in a single-blind fashion. The subjects had been asked in advance not to eat for 2 h before the experiment or drink alcohol for 24 h prior to the experiment. After drug administration, the subjects were asked to fill out two questionnaires: Swedish Universities Scales of Personality [34] and State Trait Anxiety Index–Trait [35]. Before a subject entered the MRI scanner, the rules of the UG were explained, and the subject's understanding of the game was checked with a questionnaire. All subjects passed this test.

Approximately 1 h after treatment, the first experimental session was conducted. The order in which the film clips were presented was randomized in advance, creating 18 different sequences of clips, or protocols. Each protocol, except for one, was presented for one subject receiving treatment and for one subject in the control group. We used an fMRI-compatible glove answering device in the scanner to register the subjects' responses. Subjects responded “yes” by pressing a button with their thumb and “no” by pressing a button with their index finger. All subjects underwent two scanning sessions, with a pause of approximately 1 min in between. The first session contained 23 movies, and the second session contained 22 movies.

After the scanning was completed, subjects were given a questionnaire and asked to rate the fairness of all the kinds of offers they had received, on a scale 1–7 [7]. They also viewed pictures of the proposers and rated the likeability of the faces they had seen, on a visual analog scale (0–100).

### Statistical Analyses

#### Behavioral data

The effect of the treatment on rejection rate for unfair proposals was first analyzed with a Mann-Whitney *U* test (one-tailed), since we could not assume normally distributed data. To control for stake size, sex, and ordering of decisions we analyzed the individual choices with probit regressions (not significant, see Text S1), since each individual decision is binary in nature. Standard errors were clustered on subjects to account for repeated measures. Since no fair offers were rejected, we restricted our attention to the unfair responses. Differences in ratings of fairness and likeability were analyzed with the Mann-Whitney *U* test. We used two-tailed tests, as we had no prior assumption about the direction of a potential treatment effect.

#### fMRI data

All contrasts of interest were initially analyzed on a single-subject level, and four contrasts were of interest for group data analyses. In the second-level analyses we used masks that were created with the wfu_pickatlas [36],[37] tool in SPM5. To test our main hypothesis we used a bilateral amygdala region of interest. To validate our data and perform explorative tests we also used the anatomical boundaries for the following 13 bilateral regions of interest: ACC, insula, caudate, putamen, medial orbitofrontal cortex, inferior orbitofrontal cortex, superior orbitofrontal cortex, rectus, superior medial frontal cortex, superior frontal cortex, medial frontal cortex, inferior frontal operculum, and inferior frontal triangularis. These regions of interest were selected based on the present literature [7],[24],[38],[39].

To validate the difference between an active versus passive state, proposals _unfair+fair offers_ were compared to non-proposals _control condition_ in a one-sample *t*-test including all subjects. A two-sample *t*-test comparing the two treatment groups in the same contrast was performed as a follow-up analysis. The 14-area mask (i.e., the 13-area mask including amygdala) was used in both analyses.

In our main contrast of interest, placebo _unfair−fair proposals_>oxazepam _unfair−fair proposals_, we used only a bilateral amygdala mask in order to answer our primary hypothesis and increase the sensitivity for detecting amygdala changes. The same approach was used in the second main contrast of interest, unfair proposals _rejected_>unfair proposals _accepted_. In the latter analysis we included all the subjects (*n = *6) in the placebo group that both rejected and accepted unfair proposals in both scanning sessions. In the extended analyses, 13 of the above stated regions (excluding amygdala) were used for global search in the two main contrasts of interest.

To test the main effect of unfairness within groups, we ran the contrast unfair proposals>fair proposals in the placebo group versus the oxazepam group. Moreover, to investigate gender differences, parameter estimates from right amygdala (in the contrast placebo _unfair−fair proposals_>oxazepam _unfair−fair proposals_) were analyzed with an ANOVA. Specific contrasts between gender in each treatment group were made with a between-groups *t*-test. In the correlation analyses between right amygdala activity (captured in the placebo _unfair−fair proposals_>oxazepam _unfair−fair proposals_ contrast) and rejection rate, we used a non-parametric approach (Spearman's rho). In these analyses we correlated the two variables within all subjects and in each treatment group.

In the psychophysiological interaction analyses we used right versus left amygdala as a seed region to see which other cortical regions correlated with the amygdala × model variable. Psychophysiological interaction analysis was performed first on the individual level (first level) and then on the group level (second level). In the group analyses we used the same mask as in the interaction contrast.

### Reporting Results

Only corrected results are reported in this article, with the one exception of the following contrast: placebo unfair proposals>fair proposals. All results are reported as voxel level corrected, unless otherwise stated (i.e., cluster level corrected).

### Technical Specifications for Image Acquisition and Analysis

#### Image acquisition

We used a GE 1.5T MRI scanner to measure the blood oxygen level dependent (BOLD) response. A T2* weighted echoplanar image sequence was applied. The following protocol was used: number of slices, 32; slice thickness, 4.5 mm; interslice gap, 0.5 mm; field of view, 220×220 mm; echo time, 40 ms; and repetition time, 2.5 s. A total of 168 and 161 image volumes were acquired during the two scanning sessions, respectively. In addition, we acquired an anatomical T1-weighted 3-D image volume from each subject (3D–spoiled gradient recalled), echo time/repetition time = 35/6 ms, flip = 35°, 124 coronal images, matrix size = 0.9×1.0×0.9 mm^3^).

#### Image pre-processing

The fMRI data were analyzed with SPM5 (http://www.fil.ion.ucl.ac.uk/spm/software/). The following pre-processing steps were performed: realignment, slice timing correction, co-registration, and normalization with respect to the Montreal Neurological Institute compatible echoplanar image template provided in SPM5. Finally, spatial smoothing was performed with a Gaussian kernel of 8 mm full width at half maximum. Event onset times pertaining to the proposals and control conditions were convolved with the canonical hemodynamic response function as implemented in SPM5, and inserted into a general linear model. Ten regressors were created for each scanning session to specify the model—(1) unfair proposal, (2) fair proposal, (3) no proposal (control condition)—and (4) reaction time. We corrected for residual movement-related variance in the data by including six motion parameters in the model. High-pass filtering (cutoff frequency = 128 s) was used to remove low-frequency noise. The statistical parametric map [T] map threshold was determined to *p<*0.005, uncorrected in all contrasts.

## Supporting Information

Figure S1 **fMRI results related to unfair proposals.** In the oxazepam group (*n = *17) a subsignificant activation was present in the right insula ([36 21 12], *Z* = 3.34, *p<*0.001, uncorrected).
(TIF)

Figure S2 **Neural activity related to receiving a proposal.** (A) As a manipulation check we compared proposals _unfair+fair offers_>non-proposals _control condition_ within all subjects (*n = *35). The contrast showed an activation in the frontal attention network, with a peak activation in the right mPFC (Montreal Neurological Institute space coordinates (*x*, *y*, *z*): [6 24 48], *Z* = 6.48; *p<*0.001, corrected). (B) The interaction contrast treatment _placebo>oxazepam_×proposal _unfair+fair>control condition_ showed a subsignificant activation in the left supplementary motor cortex (BA 6) ([−24 −15 57], *Z* = 4.18, *p* = 0.11, corrected).
(TIF)

Table S1 **fMRI data.** Cerebral foci of activation related to proposals and unfairness.
(DOC)

Text S1 **Supporting results and imaging data.**
(DOC)

## Abbreviations

ACC: anterior cingulate cortex
dlPFC: dorsolateral prefrontal cortex
fMRI: functional MRI
mPFC: medial prefrontal cortex
RL: reinforcement learning
SEK: Swedish crowns
UG: Ultimatum Game
