# Supplementary figures and images for: Limbic Justice—Amygdala Involvement in Immediate Rejection in the Ultimatum Game
Source: PLoS Biol. 2011 May 3;9(5):e1001054. doi: 10.1371/journal.pbio.1001054 (PMC3086869; doi:10.1371/journal.pbio.1001054)

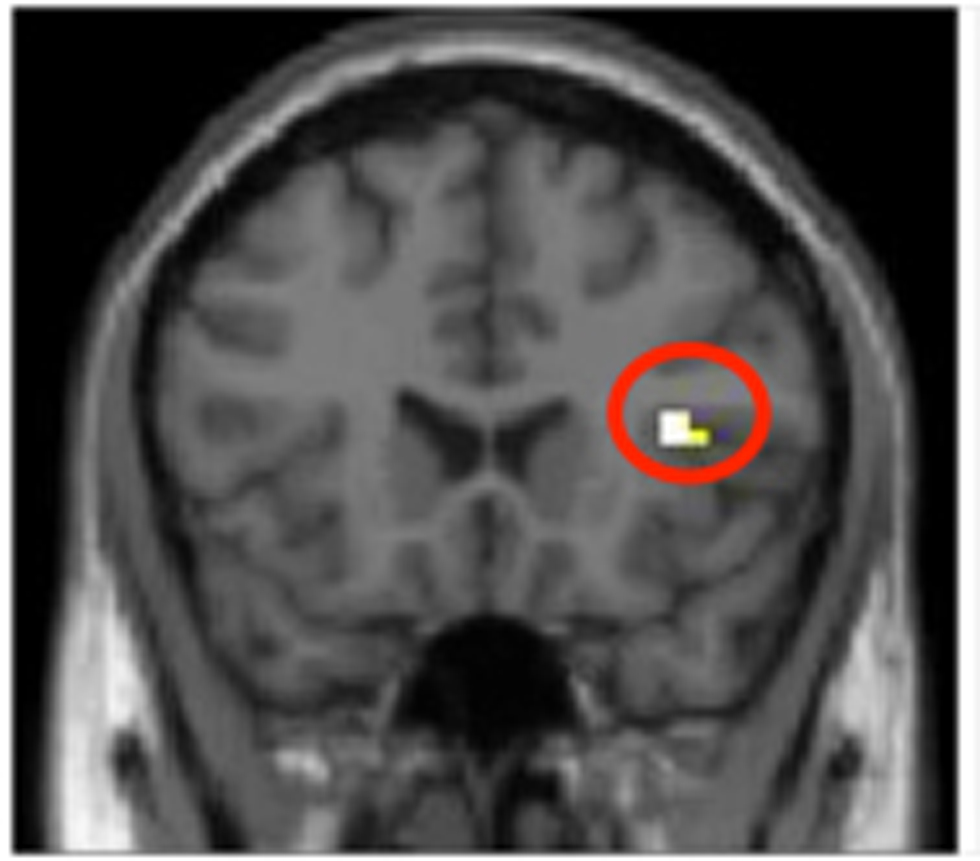

Supplement: Figure S1 — fMRI results related to unfair proposals. In the oxazepam group (n = 17) a subsignificant activation was present in the right insula ([36 21 12], Z = 3.34, p<0.001, uncorrected). (TIF) [file pbio.1001054.s001.tif]

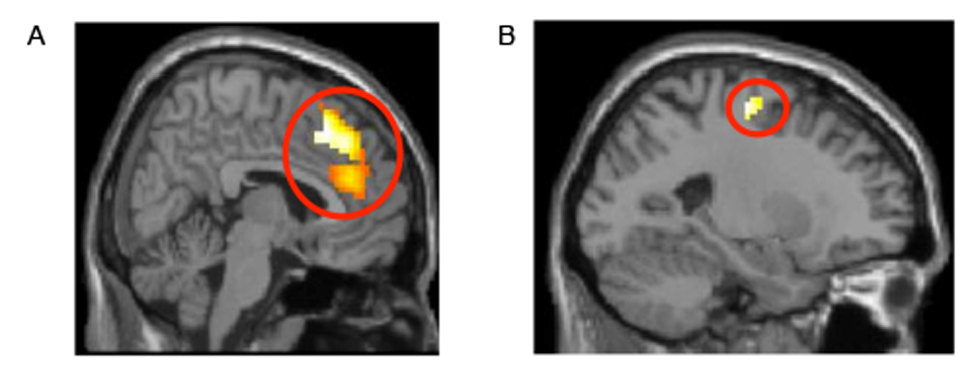

Supplement: Figure S2 — Neural activity related to receiving a proposal. (A) As a manipulation check we compared proposals unfair+fair offers>non-proposals control condition within all subjects (n = 35). The contrast showed an activation in the frontal attention network, with a peak activation in the right mPFC (Montreal Neurological Institute space coordinates (x, y, z): [6 24 48], Z = 6.48; p<0.001, corrected). (B) The interaction contrast treatment placebo>oxazepam×proposal unfair+fair>control condition showed a subsignificant activation in the left supplementary motor cortex (BA 6) ([−24 −15 57], Z = 4.18, p = 0.11, corrected). (TIF) [file pbio.1001054.s002.tif]
