# Supplementary material for: Limbic Justice—Amygdala Involvement in Immediate Rejection in the Ultimatum Game
Source: PLoS Biol. 2011 May 3;9(5):e1001054. doi: 10.1371/journal.pbio.1001054 (PMC3086869; doi:10.1371/journal.pbio.1001054)
Supplement: Table S1 — fMRI data. Cerebral foci of activation related to proposals and unfairness. (DOC) [file pbio.1001054.s003.doc]

# Supporting table

**Table S1. fMRI data.** Cerebral foci of activation related to proposals and unfairness.

| Contrast | Region | Hemi | Correction (p < ) | # Voxels | MNI space coordinates | | | Z score |
| --- | --- | --- | --- | --- | --- | --- | --- | --- |
|  |  |  |  |  | x | y | z |  |
| (p > np) _all subjects_ | SMA | R | 0.000 cluster-level | 2973 | 6 | 24 | 48 | 6.40 |
|  | Precentral gyrus | L | 0.000 cluster-level | 1731 | -45 | 3 | 27 | 6.25 |
|  | Precentral gyrus | L | 0.000 cluster-level | 144 | -36 | 0 | 57 | 4.75 |
|  | Caudatus | R | 0.000 cluster-level | 254 | 12 | 15 | -3 | 4.45 |
| (p > np) _placebo_ > (p > np) _oxazepam_ | SMA | L | 0.11 voxel-level | 25 | -24 | -15 | 57 | 4.18 |
| (u > f) _placebo_ | ACC | R | 0.001^†^ voxel-level | 66 | 9 | 48 | 24 | 3.15 |
|  | dlPFC | L | 0.001^†^ voxel-level | 12 | -24 | 36 | 54 | 4.04 |
|  | dlPFC | R | 0.001^†^ voxel-level | 54 | 30 | 36 | 51 | 4.04 |
|  | Insula | L | 0.01^†^ voxel-level | 5 | -30 | 24 | 3 | 2.90 |
| (u > f) _oxazepam_ | Insula | R | 0.001^†^ voxel-level | 13 | 36 | 21 | 12 | 3.34 |
| (u > f ) _placebo_ > (u > f ) _oxazepam_ * | Amygdala | L | 0.05 voxel-level | 9 | -18 | -6 | -18 | 3.25 |
|  | Amygdala | R | 0.05 voxel-level | 3 | 18 | 0 | -18 | 3.03 |
| (u > f ) _placebo_ > (u > f ) _oxazepam_ ** | mPFC | L | 0.035 cluster-level | 143 | -6 | 66 | 18 | 3.77 |
|  | mPFC | L |  |  | -9 | 51 | 24 | 3.74 |
|  | ACC | R |  |  | 9 | 48 | 24 | 3.57 |

p = proposal; np = no proposal; u = unfair; f = fair; SMA = supplementary motor area; ACC = anterior cingulate cortex; dlPFC = dorsolateral prefrontal cortex; mPFC = medial prefrontal cortex; ^†^ = uncorrected; Hemi = hemisphere; R = right; L = left. * = primary analysis. ** = explorative analysis.
