# Supplementary material for: Limbic Justice—Amygdala Involvement in Immediate Rejection in the Ultimatum Game
Source: PLoS Biol. 2011 May 3;9(5):e1001054. doi: 10.1371/journal.pbio.1001054 (PMC3086869; doi:10.1371/journal.pbio.1001054)
Supplement: Text S1 — Supporting results and imaging data. (DOC) [file pbio.1001054.s004.doc]

# Supporting information

## Supporting results

### Behavioral data for rejection rate

### Gender differences

Neither the rejection rate nor the effect of treatment on the rejection rate did vary significantly between men and women. Men’s average rejection rate dropped by 19.6 percentage points as an effect of treatment, from 30.7 % to 11.1 % (Mann-Whitney U test, one-tailed, Z = 1.036, p = 0.150). Women’s average rejection rate dropped by 15.1 percentage points by treatment, from 40.4 % to 25.3 % (Mann-Whitney U test, one-tailed, Z = 1.116, p = 0.132).The effect of treatment was significant in a probit regression analysis on the rejection rate (one-tailed test, Z = -1.668, p = 0.047) and the predicted reduction in the rejection rate due to treatment was 18.3 percentage points (the predicted rejection rate was 37.5 % in the oxazepam group and 19.2 % in the placebo group). When adding gender to this regression analysis and testing whether gender affects the rejection rate, gender was not significant (two-tailed test, Z = 1.086, p = 0.278). The significance of the treatment effect decreased somewhat in this probit regression (one-tailed test, Z = -1.493, p = 0.067), and the predicted reduction in the rejection rate due to treatment decreased from 18.3 percentage points to 16.7 percentage points (the predicted rejection rate was 36.14 % in the oxazepam group and 19.46 % in the placebo group). To test if the effect of treatment varied between men and women we also added an interaction variable for gender and treatment in addition to the gender dummy variable. This interaction variable was not significant (two-tailed test, Z = 0.414, p = 0.679). We also tested the joint significance of the gender dummy variable and the interaction variable using a Wald test, but these variables were not jointly significant (Chi-square (2 df), p = 0.467).

### Robustness to stake effects

Stake levels did not significantly influence the rejection rate. To test if the rejection rate was affected by stakes we added two dummy variables for the SEK 250 and the SEK 500 stakes in a probit regression. These dummy variables were not significant (two-tailed test of the SEK 250 variable, Z = -0.199, p = 0.842; two-tailed test of the SEK 500 variable, Z = -0.528, p = 0.597). The effect of treatment on the rejection rate remained after controlling for the different stake levels, in the probit regression (one-tailed test, Z =1.668, p=0.047). Gender was not significant when added to this regression equation (two-tailed test, Z=1.084, p=0.279). Adding also the interaction between gender and treatment did not significantly improve the model (two-tailed test of the interaction coefficient, Z=0.407, p=0.684; Wald test of the joint significant of the gender dummy and the interaction, Chi-square (2 df), p=0.468).

### Robustness over time

To test if rejection rate changed over time we conducted a probit regression where we included the order of the decision (1-45) as an explanatory variable, together with dummy variables for the stake levels. The order variable was not significantly related to the rejection of unfair offers (two-tailed test, Z = -0.988, p = 0.323), and it did not affect the treatment effect (one-tailed test, Z = -1.668, p = 0.047). A similar result was observed when a dummy variable for the second half of the films was used as an explanatory variable (two-tailed test, Z = -0.478, p = 0.633) and the treatment effect was not affected here either (one-tailed test, Z = -1.670, p = 0.047). Gender was not significant when added to either of these two regression equations (two-tailed test in the first regression, Z = 1.079, p = 0.281; two-tailed test in the second regression, Z = 1.083, p = 0.279). Adding also the interaction between gender and treatment did not significantly improve the models either (two-tailed test of the interaction coefficient in the first regression, Z = 0.404, p = 0.686; two-tailed test of the interaction coefficient in the second regression, Z = 0.407, p = 0.684; Wald test of the joint significant of the gender dummy and the interaction in the first regression, Chi-square (2 df), p = 0.472); Wald test of the joint significant of the gender dummy and the interaction in the second regression, Chi-square (2 df), p = 0.469).

## Imaging data

### Main effect of proposals

To test the validity of the study design we used the contrast proposals _unfair + fair offers_ > non proposals _control condition_ within all subjects as an overall check of the subject’s attention. The analysis showed strong activations bilaterally in the frontal attention network with a peak activation in the right medial prefrontal cortex (mPFC) (MNI space coordinates (x,y,z): [6 24 48] Z = 6.48; p < 0.001 corrected) (Fig. S2A), providing strong support that our subjects indeed actively participated in the experiment. Moreover, we were interested in excluding the possibility that the oxazepam treatment had a general effect on neural activity. Therefore, we tested the interaction placebo unfair+fair proposals – control condition > oxazepam unfair+fair proposals – control condition. This contrast only showed a subsignificant activity in the left supplementary motor cortex (BA 6) (peak voxel: [-24 -15 57] Z = 4.18, p = 0.11 corrected) (Fig. S2B) possibly reflecting the preparation to make an active choice. In summary, we could conclude that the effect seen in Figure S2A was driven by both groups and not by the placebo group alone. Thus, the general cerebral response in the decision making task was unaltered by the drug.

### Rejection rate and amygdala activity

Rejection rate for unfair proposals and (right) amygdala activity as measured in the placebo _unfair – fair proposals_ > oxazepam _unfair - fair proposals_ contrast did not correlate significantly when all subjects were included in the analysis (r = 0.070, p = 0.34, one-tailed). Similarly, the within group correlation analyses did not yield any significant results (placebo: r = 0.25, p = 0.17, one-tailed; oxazepam: r = 0.21, p = 0.20, one-tailed).

### Rejected unfair proposals vs. accepted unfair proposals

For completeness we performed a post hoc analysis over all subjects (n = 21) that rejected unfair proposals in either the first or second session or both. Treatment and gender were added as covariates of no interest in the analysis. The contrast: unfair proposals _rejected_ > unfair proposals _accepted_ yielded a subsignificant result in right amygdala ([21 -3 -18] Z = 2.95, p = 0.065).

### Controlling for gender effects in the interaction contrast

When gender was added as a factor of no interest to the GLM model the significance still remained in the interaction contrast placebo unfair – fair proposals > oxazepam unfair - fair proposals (left amygdala: Z = 3.08, p = 0.035; right amygdala: Z = 3.02, p = 0.042). This indicates that our observation in the interaction contrast is not a gender effect, but an effect of treatment.

### The insula

Although we did not observe any significant interaction for (unfair vs. fair proposals) placebo vs. (unfair vs. fair proposals) oxazepam we present detailed data on the insula since it has been suggested that this structure is associated to rejection of unfair proposals [8]. The main effect of unfairness (unfair – fair proposals) in the oxazepam group showed a subsignificant activation in right insula ([36 21 12] Z = 3.34, p < 0.001 uncorrected) (Fig. S1). The same contrast in the placebo group showed a subsignificant activation in the left insula ([-30 24 3] Z = 2.90, p < 0.005 uncorrected). These Z-scores are in the same range as the significance values presented for insula activation in a previous imaging study of the ultimatum game [8] however, we implemented a more conservative statistical correction procedure.

Since we performed a correction involving a 13-region ROI mask for the insula analysis there is a risk for a typ-2 error. Therefore we also performed an explorative analysis using a specific ROI for insulae in the interaction analysis (placebo _unfair – fair proposals_ > oxazepam _unfair - fair proposals_). This analysis did not yield any significant results (threshold p < 0.005). However, when using a lower threshold of p < 0.05 an insula activation (p = 0.001) was detected on set level [peak voxel: X Y Z = 33 0 12, Z = 2.48]. Thus, although we did not observe any robust difference in insula between the two treatment groups in processing unfair proposals (placebo _unfair – fair proposals_ > oxazepam _unfair - fair proposals_) its involvement in this effect cannot be excluded.

### The dorsolateral prefrontal cortex

Dorsolateral prefrontal cortex has been suggested to have an important role for decision making. Therefore, we choose to explore this region with a specific mask (BA 9/46) when we did not find any specific activation in the interaction contrast (as for insula). A threshold at p < 0.005 yield a significant result on set level [X Y Z = -3 51 21, Z = 3.51, p = 0.036]. Thus, it cannot be excluded that the effect of benzodiazepines also acts on this structure.

### Psychophysiological interaction analyses

Our paradigm was primarily designed to catch short term events and therefore not optimal for functional connectivity analyses. However, for a matter of completeness we investigate if any cortical regions covaried with changes in amygdala activity in the unfair condition compared to the fair condition using psychophysiological interaction (PPI) analyses. In the placebo group we found no significant PPI between amygdala and other brain regions whereas in the treatment group we observed a trend PPI between left amygdala and right insula ([27 -12 12]; Z = 3.51, p = 0.151, cluster level corrected). In the oxazepam _unfair - fair proposals_ > placebo _unfair – fair proposals_ contrast there was a trend PPI between right amygdala and left dlPFC ([-45 21 0]; Z = 3.17, p = 0.069, cluster level corrected). Thus, the data suggests that the functional relation between amygdala and insula/dlPFC may change as a consequence of fairness and treatment. However, the relation seems to be more complex than an increased connectivity in unfair condition between amygdala and cortical regions that is suppressed after treatment with benzodiazepine agonists. Optimally, further functional connectivity analysis of UG should be performed in block design studies.
